# Insecticidal Activity and Insecticidal Mechanism of Total Saponins from *Camellia oleifera*

**DOI:** 10.3390/molecules24244518

**Published:** 2019-12-10

**Authors:** Chuanjian Cui, Yunqin Yang, Tianyu Zhao, Kangkang Zou, Chuanyi Peng, Huimei Cai, Xiaochun Wan, Ruyan Hou

**Affiliations:** State Key Laboratory of Tea Plant Biology and Utilization, Key laboratory of Food Nutrition and Safety, School of Tea and Food Science & Technology, Anhui Agricultural University, Hefei 230036, Chinayangyunqiu@ahau.edu.cn (Y.Y.); pcy0917@ahau.edu.cn (C.P.); hml20@sina.com (H.C.); xcwan@ahau.edu.cn (X.W.)

**Keywords:** tea saponin, *Camellia oleifera*, *Ectropis obliqua*, contact toxicity, stomach toxicity, waxy layer, insecticidal mechanism

## Abstract

Chemical pesticides are commonly used during the cultivation of agricultural products to control pests and diseases. Excessive use of traditional pesticides can cause environmental and human health risks. There are ongoing searches for new plant-derived pesticides to reduce the use of chemical pesticides. In this study, tea saponin extracts of different purities were extracted from *Camellia oleifera* seeds using AB-8 macroporous resin and gradient elution with ethanol. The insecticidal effects of the tea saponin extracts were evaluated by contact toxicity tests and stomach toxicity tests using the lepidopteran pest of tea plantation, *Ectropis obliqua*. The total saponins extracted using 70% ethanol showed strong contact toxicity (LC_50_ = 8.459 mg/L) and stomach toxicity (LC_50_ = 22.395 mg/L). In-depth mechanistic studies demonstrated that tea saponins can disrupt the waxy layer of the epidermis, causing serious loss of water, and can penetrate the inside of the intestine of *E. obliqua*. After consumption of the tea saponins, the intestinal villi were shortened and the cavities of the intestinal wall were disrupted, which resulted in larval death. This study highlights the potential of tea saponins as a natural, plant-derived pesticide for the management of plant pests.

## 1. Introduction

Tea is one of the most popular non-alcoholic beverages in the world today, favored for its unique aroma and taste [1]. *Ectropis obliqua* Prout (Lepidoptera:Geometridae) is a chewing defoliator in tea plantations, causing devastating effects on the quality, yield, and growth of tea plants [2,3]. Because of its fast breeding, rapid spatial spread, and large appetite, outbreaks of this insect have resulted in losses of more than 60% in tea production [4]. *E. obliqua* is the primary target pest in tea cultivation [5].

At present, *E. obliqua* is mainly controlled by chemical pesticide spraying [6]. Tea is a direct receptor of aerial pesticides, and pesticide residues may cause food safety and other issues. At the same time, studies have shown that only a small amount (about 0.3%) of pesticides can enter the target organism and that most pesticides (99.7%) will eventually enter the environment, posing environmental safety hazards [7]. International trade standards demand higher quality agricultural products and set maximum residual levels of pesticides in tea products. Plant-derived products can be more environmentally friendly and can represent cost-effective alternatives to control phytophagous insects and plant-pathogenic microorganisms. Therefore, biological control of this pest has become the method of choice [5]. 

Tea saponins (TS) are a promising bio-pesticide with good dispersibility, permeability and wetting effect [8]. Tea saponin content in the seeds of *Camellia oleifera* is greater than 10%. The annual output of *C. oleifera* has exceeded 2 million tons, and about 200,000 tons of saponins can be supplied for use [9]. Previous studies have found that tea saponins have a strong insecticidal effect on the diamondback moth, *Plutella xylostella,* and aphid, *Aphis craccivora* [10]. In a study on saponin repellent activity, saponins at 4000 mg/L highly repel (48.57%) the third instar of *P*. *xylostella*. However, the feeding preference index (PI) of the third instar for saponins was less at higher concentrations (0.63). Treatment of diamondback moth larvae with LC_20_ and LC_50_ doses of TS led to lower growth rates, decreased feed consumption, reduced frass production, lower pupal weights, reduced percentage pupation, slower adult emergence, and diminished fecundity, but prolonged durations of the larval and pupal periods [10]. 

Most current research indicates that the insecticidal mechanism of tea saponins is related to effects on the detoxification enzymes of insects. Tea saponins can reduce superoxide dismutase (SOD), catalase (CAT), acetylcholinesterase (AChE), and carboxylesterase (CES) activities [11,12]. Some studies suggest that the insecticidal activity of saponins is due to their interaction with cholesterol, which leads to interference with the synthesis of ecdysteroids. These substances are also protease inhibitors or cytotoxic to certain insects [13]. However, the specific insecticidal mechanism has not been reported.

In this paper, we isolated saponin extracts from *C*. *oleifera* seeds for the purpose of developing new plant-derived pesticides. Using the common pest *E*. *obliqua* as a model, the insecticidal effect of tea saponins was comprehensively evaluated by contact toxicity and stomach toxicity tests. The insecticidal mechanism of tea saponins against *E*. *obliqua* larvae was studied microscopically, and the source of its insecticidal activity was found.

## 2. Results

### 2.1. Saponins in Aqueous Ethanolic Extracts

Tea saponins were extracted from *Camellia oleifera* seeds using ethanol and then purified using AB-8 macroporous resin and a gradient ethanol elution. The obtained eluates were subjected to thin-layer chromatography in combination with a sulfuric-acid–ethanol color reaction (Figure S1). The purple color indicating saponins was observed in different eluents. Of all the eluates, 70% ethanol eluate (EE) did not have a chromogenic reaction of flavonoids, which demonstrates the higher purity of the saponin in the 70% ethanol eluate.

The extracted tea saponins were analyzed by HPLC (Figure S2). Since there are no commercial *Camellia* saponin standards, the total saponin content was quantified using an individual oleiferasaponin C1 standard, as described previously [14]. The oleiferasaponin C1 standard curve was expressed as *y* = 0.6938*x* − 1.5247 (R^2^ = 0.9999), showing a linear correlation over the concentration range of 0.005 to 1.0 mg/mL. 

The purity of the saponins extracted using different ethanol–water eluents was tested using a standard multitest method. The purity of the saponins in the *C*. *oleifera* seed cake ethanol extract (CSCEE) was 39.5% ± 3.46%; in the 30% EE, the purity was 35.9% ± 2.31%; in the 50% EE, the purity was 55.6% ± 1.69%; in the 70% EE, the purity was 99% ± 0.71%. 

UPLC-Q-TOF/MS analysis was used to characterize the saponins in the 70% EE. The eluate contained 29 triterpenoid saponins (Figure S3). The molecular formulas and molecular weights of these saponins were confirmed by high-resolution mass measurements, deprotonated molecular ions [M − H]^−^, isotope abundance for each pseudomolecular ion, and fragment ions (Tables S1 and S2). The identification of each saponin was based on published fragmentation data and nominal masses calculated from known structures [8,14]. 

### 2.2. Dynamic Viscosity Coefficient and Interfacial Tension Values of Different Extracts

**Figure 1:**
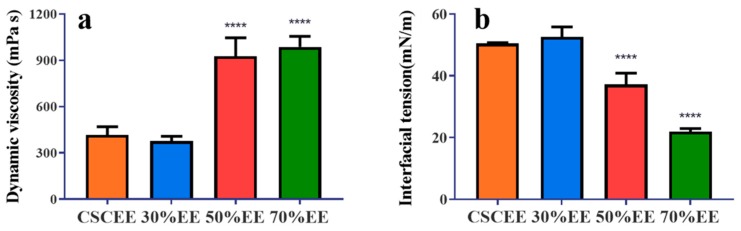
Viscosity coefficient and surface tension of the original seed cake extract and the different eluates from the AB-8 macroporous resin. (**a**) Viscosity coefficient; (**b**) surface tension (CSCEE, *C*. *oleifera* seed cake ethanol extract; 30%EE, 30% ethanol eluate; 50%EE, 50% ethanol eluate; 70%EE, 70% ethanol eluate). Asterisks (****) indicate significant differences compared with *C*. *oleifera* seed cake ethanol extract (*p* < 0.05), *n* = 5, average ± SD. ANOVA with Tukey’s HSD post-hoc test.

The dynamic viscosity coefficients of the four saponin extracts were evaluated (Figure 1a). There was no significant difference in the kinematic viscosity coefficient of the 30% EE (376.8 MPa·s) and the CSCEE (415.6 MPa·s). Both the 50% EE (925 MPa·s) and the 70% EE (968.4 MPa·s) showed significantly higher kinematic viscosity coefficients than the seed cake ethanol extract.

The interfacial tension values of the four saponin extracts were measured after 1000-fold dilution (Figure 1b). The results showed that the surface tension of the 70% EE (21.86 mN/m) was significantly lower than that of CSCEE (50.412 mN/m), the 30% EE (52.64 mN/m), and the 50% EE (37.18 mN/m).

### 2.3. Toxicty of the Saponins on the Stomach of E. obliqua Larvae

All saponin extracts were evaluated for toxicity to the stomach of *E*. *obliqua* larvae. No individuals died when fed distilled water (control) within 48 h, so no adjustment for corrected mortality was necessary. The insecticidal effect of the 70% EE (LC_50_ = 22.395 mg/L) was significantly higher than that of CSCEE (LC_50_ = 49.100 mg/L, *p* < 0.05), 30% EE (LC_50_ = 53.239 mg/L, *p* < 0.05) and 50% EE (LC_50_ = 45.287 mg/L, *p* < 0.05) (Table 1).

### 2.4. Contact Toxicity of the Camellia Saponins on E. obliqua Larvae

Using the protocol for testing pesticides against *Myzus persicae* (Sulzer), the contact toxicity of the four saponin extracts on *E*. *obliqua* larvae were compared [15]. Overall, the toxicity of the four saponins was higher via contact than in the stomach (Table 2). The insecticidal activities were further evaluated by calculating the LC_50_ values of the four treatments on *E*. *obliqua*. The LC_50_ value of the 70% EE was only 8.459 mg/L, significantly lower (at *p* < 0.05) than that of the CSCEE (LC_50_ = 27.380 mg/L), the 30% EE (LC_50_ = 21.004 mg/L), and the 50% EE (LC_50_ = 15.732 mg/L).

### 2.5. Midgut of E. obliqua Larvae and Morphological Changes after Tea Saponin Treatment

**Figure 2:**
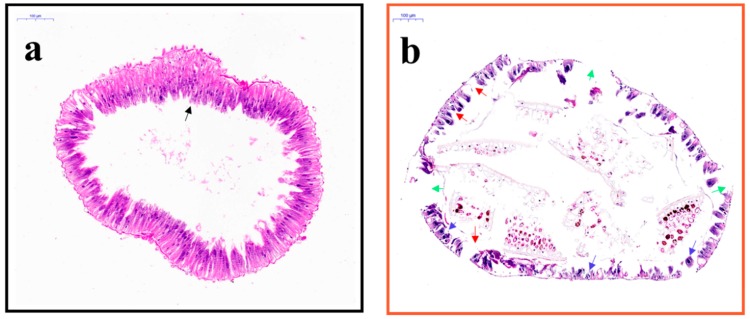
Cell staining of the intestinal tract of the *E. obliqua* larvae and the structural changes after (**a**) water treatment and (**b**) tea saponin treatment.

Larvae were treated for saponin for 24 h, and the midgut was sectioned and observed by transverse staining. In the control group (Figure 2a), the midgut epithelium consisted of a single layer of digestive cells exhibiting a well-developed brush border and cytoplasm with acidophilic regions (Figure 2a, black arrow indicates example section). In contrast (Figure 2b), exposure of *E*. *obliqua* larvae to tea saponins resulted in a cell apex with a damaged brush border (Figure 2b, red arrow sections) and nucleus with decondensed chromatin and evident nucleoli (Figure 2b, blue arrow section). The intestinal wall was disrupted and had several holes (Figure 2b, green arrows).

### 2.6. Observation of the Epidermis of E. obliqua Larvae Treated with Tea Saponin by Electron Microscopy 

**Figure 3:**
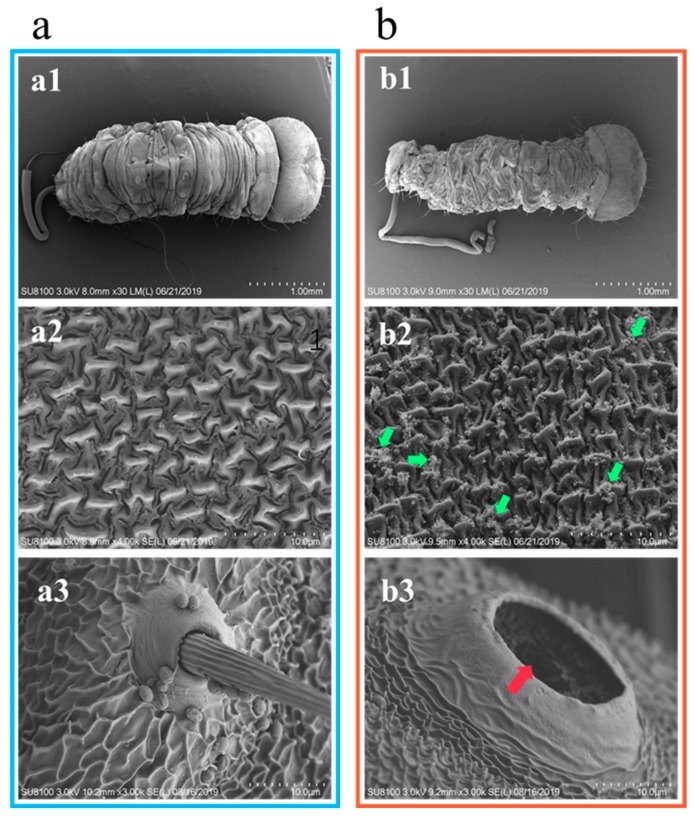
Scanning electron microscopy images of surfaces of *E*. *obliqua* larvae. (**a**) Control treatment at 100×(a1), 5000× (a2), and 5000× (a3) near the hairs. (**b**) Tea saponin treatment at 100×, 1000× and 5000× (b1, b2, and b3 respectively).

The effects of the two treatments on the epidermis of the *E*. *obliqua* larvae were observed by scanning electron microscopy (Figure 3). Compared with the control treatment (Figure 3a, panel a1), the epidermis layer of the *E*. *obliqua* larvae treated with the aqueous solution containing tea saponins became rough and formed severe wrinkles (Figure 3b, panel b1), and the trunk portion was dehydrated and shrunk (magnification at 100×). The waxy layer of the epidermis was ablated (at 5000×, Figure 3b, panel b2, green arrow section) and voids appeared in the epidermis (Figure 3b, panel b3, red arrow section). 

### 2.7. Chitin Staining of E. obliqua Larvae and Morphological Changes after Tea Saponin Treatment

**Figure 4:**
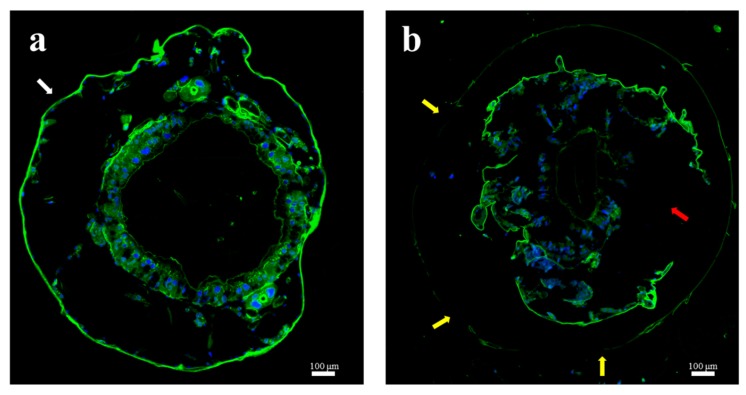
Chitin staining of *E. obliqua* larvae and midgut sections: (**a**) control; (**b**) tea saponin treatment).

In order to investigate the cause of the epidermal damage after tea saponin treatment, the chitin layer of the *E*. *obliqua* larvae was stained with lectin (Figure 4). Compared with the control (Figure 4a), the tea-saponin-treated *E*. *obliqua* showed thinning of the epidermal chitin and ablation (Figure 4b, yellow arrow), and the intestine (enclosed by the chitin layer) was ulcerated (Figure 4b, red arrow).

## 3. Discussion

In the search for novel plant-derived compounds for pest treatment in tea gardens, we found that a saponin extract from the same *Camellia* genus has advantages as an insect feeding deterrent and insecticide. In previous studies, tea saponins have been reported as natural, triterpenoid-derived compounds with insecticidal activity and resistance to insects [8,11]. Chen et al. (1996) demonstrated that a solution containing 25% active tea saponins significantly increased the larval mortality (84%) of the cabbage butterfly (*Pieris rapae* Linne) [16]. In this study, the classic stomach toxicity test was used to evaluate stomach toxicity of tea saponins of different purities on *E*. *obliqua* larvae [17]. The results further verified that tea saponins have insecticidal properties and tea saponins with 99% purity showed 2.2-fold higher stomach toxicity than did crude ethanol extract on *E*. *obliqua* larvae. The purity of tea saponins in the extract showed a good linear relationship with the insecticidal toxicity (R^2^ = 0.9865), which proved that tea saponins are the main insecticidal active ingredients in *C. oleifera* seed extract [18,19]. 

In this study, tea-saponin-treated leaves caused severe damage to the midgut of *E*. *obliqua* larvae. The main functions of the midgut include the production of digestive enzymes and nutrient uptake of digested products [20,21,22,23]. The peritrophic matrix (PM) in the midgut of insects consists primarily of chitin and proteins and is thought to support digestion and provide protection from abrasive food particles and enteric pathogens [24]. The results indicate that tea saponins can cause physiological and morphological damage to midgut epithelial cells. After absorption of tea saponins, the cells exhibited vacuolization and vesicle release for energy detoxification. Excessive toxicity of tea saponins leads to smaller microvilli in the midgut and to cell death. 

Evaluation of the contact toxicity of natural plant-derived extracts is important [25,26]. Some natural products have been reported to have direct contact toxicity on insects. For instance, linalool exhibited contact toxicity with an LC_50_ of 105.63 μg/cm^2^ against *S*. *oryzae* and *Tribolium castaneum* [27]. Wang et al. found that the essential oil from *Z. purpureum* rhizomes possessed strong contact toxicity against *T*. *castaneum* and *Lasioderma serricorne* adults, with LD_50_ values of 39.0 and 16.3 µg per adult, respectively [28]. However, the contact toxicity of tea saponins has rarely been reported [29]. During these experiments, we found that an aqueous tea saponin solution can more easily adhere than just water to the back epidermis of *E*. *obliqua*. A high kinematic viscosity coefficient is beneficial to the effective adhesion of pesticides to the target insects and crops during spraying, and is positively correlated with the utilization rate of pesticides [30]. Our results are consistent with this, as the 70% EE saponin solution has a higher viscosity coefficient and a better contact toxicity against *E*. *obliqua*. This property could limit the movement of the larvae to some extent while increasing the effective retention time of the droplets on its epidermis. 

The epidermis (exoskeleton) of *E*. *obliqua* is composed of a waxy layer and a chitin layer. The waxy later is the outermost layer of the epidermis and is highly hydrophobic. It is difficult for pesticides to penetrate into the skin or even the body. Therefore, it is important to evaluate the interfacial tension of the pesticide droplets. The lower the interfacial tension, the faster the pesticide droplets can spread on and penetrate the insect surface, thus improving the bioavailability of the pesticide [31,32]. In this experiment, the 70% EE had the lowest interfacial tension and the strongest contact effect on *E*. *obliqua*. The purity of the tea saponin showed a good linear relationship with the interfacial tension (R^2^ = 0.9598). At the same time, the interfacial tension and contact toxicity also showed a good linear relationship (R^2^ = 0.9136). These data proved that tea saponins can effectively adhere to and penetrate into *E*. *obliqua* and that the high viscosity and low interfacial tension of tea saponins result in a higher bioavailability than some other natural products.

Some studies suggest that the waxy layer is the primary barrier that protects insects from external compounds [33]. The waxy layer prevents dryness, perceives the environment, and provides mechanical support and movement. In high-chlorine and octyl oil solutions, the waxy layer of the insect body is destroyed by adding a high-efficiency wax penetrant, so that the active ingredient penetrates the surface of the body and reaches the target site, thereby generating strong contact toxicity [34]. Some natural products have been found to disrupt the insect waxy layer, but the effects of saponins on insect waxy layers have rarely been reported [35]. In the present study (Figure 3), observation by scanning electron microscopy showed that a large area of ablation occurred in the waxy layer of the *E*. *obliqua* treated with tea saponins, resulting in the appearance of pores on the larvae and loss of water. The results revealed that tea saponins can cause higher toxicity during a short contact time. Tea saponins affect the water balance of the larvae by destroying the waxy layer on the surface, allowing penetration into the body and finally resulting in death due to water loss.

Many studies have shown that the thickening of the chitin layer is the main mechanism for insects to improve pesticide resistance. For example, a population of *Anopheles gambiae* originating from West Africa is resistant to pyrethroids and DDT due to a thickening of all chitin layers (exocuticle, mesocuticle and endocuticle) [36]. The proteins CPLCG3 (and G4) are expressed at higher levels in pyrethroid-resistant compared to susceptible mosquitoes [37,38]. Localization of these proteins in limbs and particularly in the endocuticle is consistent with the cuticle-thickening resistance mechanism [39]. Previous studies have shown that tea saponins can significantly induce chitinase activity in mycelial cells, causing chitin hydrolysis, resulting in increased cell membrane permeability as well as leakage of soluble proteins and reduction of reducing sugars [13,40]. Due to their amphiphilic structures, tea saponins easily interact with cholesterol substances and can interfere with the synthesis of ecdysteroids, thereby causing damage to the insect epidermis. In *E*. *obliqua*, chitin staining showed that the chitin content of the outer epidermis was significantly reduced after treatment with tea saponin and that the intestine disrupted after breakdown of the chitin in the intestinal tract. This proved that tea saponins can destroy not only the waxy layer on the surface of *E*. *obliqua*, but also the chitin layer both outside and in. 

Together these results confirm that tea saponins have a strong insecticidal effect on the larvae of the tea plantation pest *E*. *obliqua*. The saponins can destroy the outer surfaces of the larvae by contact and can destroy the intestines following consumption. Through two different mechanisms of action, tea saponins can effectively control *E*. *obliqua* and protect tea trees. As a plant-derived insecticidal compound, tea saponins have a great potential to become a green pesticide, while future research will further explore the synergy of tea saponins with other types of pesticides (including biological pesticides) for the development of better pesticides.

## 4. Materials and Methods 

### 4.1. Plant Material and Extraction

Camellia oleifera seed cake (20 kg, Yuzigui Oil Tea Co., Ltd., Huangshan, China) was dried, pulverized, and extracted twice with 70% ethanol at 60 °C for 2 h. The ethanol extract solution was filtered and dried with a spray dryer (LABMAQ, Riberão Preto-SP, Brazil, model MSDI 1.0). The extract powder was diluted to 3% (*w*/*v*) with deionized water and batch-loaded onto a column (Chemical Co., Ltd., Xuzhou, Jiangsu, China,) containing AB-8 macroporous resin. After loading of the sample, the column was eluted with water (16 L) followed by increasing amounts of ethanol, beginning at 30% (16 L), 50% (16 L), and then 70% (32 L). The elutes were separately collected and dried for later analysis.

### 4.2. Analytical Method

The sulfuric-acid–ethanol color reaction was used for quality assessment of the tea saponin eluates. The ethanol extract of the tea saponin was diluted to 10 mg/mL. Aliquots of the saponin solution were spotted on the TLC plates (pre-coated 20 cm × 20 cm and 0.25 mm thickness, Anhui Liangchen Silicon Source Material Factory) using a capillary tube. Each sample-spotted sheet was put into a solvent system using toluene–ethyl-acetate–water (4:1:0.7, *v*/*v*/*v*) as the mobile phase for a 5-min immersion. The plate was moved from developing cylinder and dried in an oven at 120 °C for 3 min. The plate was sprayed uniformly with 10% sulfuric acid-ethanol-vanillin solution, and then put on an iron plate at 105 °C for 5 min for the color change. The reaction of tea saponins with sulfuric-acid–ethanol would appear purple, while flavonoids would be yellow. 

The purity of the tea saponin samples was further detected with HPLC according to the published method [14]. The oleiferasaponin C1 standard was accurately weighed (5 mg), sonicated with methanol, and diluted to 1 mL to yield a standard stock solution [41]. This was diluted to different concentrations with methanol to yield standard working solutions of 1000, 500, 200, 50, and 20 mg/L. High-performance liquid chromatography (HPLC) (Agilent1260, Santa Clara, CA, USA) was used to detect the purity of the *C*. *oleifera* seed cake ethanol extract (water elution, CCEE), 30% ethanol eluate (EE), 50% EE, and 70% EE. The 70% EE was further evaluated by ultrahigh-performance chromatography coupled with electrospray ionization quadrupole time-of-flight mass spectrometry (UPLC-Q-TOF/MS), as described in [14].

### 4.3. Interfacial Tension and Dynamic Viscosity Coefficient of the Different Extracts

The viscosities of the extracts were first estimated to select the appropriate speed (12 RPM) and rotor size (L2) for the NDJ-8S digital viscometer (Shanghai Pingxuan Scientific Instrument Co., Ltd., Shanghai, China). The four extracts were diluted with deionized water 1000 times. After 2 min of equilibration at 25 °C, the measurement was repeated 5 times. Data are expressed in MPa·s.

The interfacial tension of the extract was determined using the maximum bubble method, with water as the constant for the liquid to be measured (DMPY-2C, Nanjing Nandawanhe Technology Co., Ltd., Nanjing, China). The end-point of the capillary was tangent to the water surface. The pesticide drop rate from the capillary was set to one drop every 5 to 10 s. Each time a bubble escaped, the maximum pressure difference was recorded. Recordings were made for 10 to 20 groups to determine the average ΔP_max_ of water. The surface tension of the water at the experimental temperature was determined, and the instrument constant, K, was calculated by the formula K = ΔP_maxwater_/σ_water_. To determine the surface tension of the sample, the test and capillary tubes were washed with the solution to be tested before an appropriate amount of sample was added to the test tube for measurement according to the instrument constant. The maximum pressure difference between 10 and 20 groups when jumping out of the bubble was recorded. Substitution of the average into the formula K = ΔP_maxwater_/σ_water_ = Δ P_maxmeasured_/σ_measured_ was used to calculate the surface tension.

### 4.4. Bioassays

#### 4.4.1. Evaluation of the Contact Toxicity of *Camellia* Saponins on *E. obliqua*

A total of 2000 *E. obliqua* at the second instar stage were randomly divided into groups. The untreated (blank) group consisted of 80 *E. obliqua*. The remaining *E. obliqua* were treated with *C. oleifera* seed cake ethanol extract, 30% EE, 50% EE, and 70% EE. Each pesticide treatment was diluted with deionized water to 8 different concentrations (5, 10, 15, 20, 25, 30, 40, and 50 mg/L). Twenty *E. obliqua* larvae were tested as a group within the experiments, and three biological replicates were performed. A Burkard automated microtiter was used to drop 1 μL of a treatment onto the back of each larvae. Mortality was observed 24 h after treatment and used to establish the respective concentration-response curves. The experiment was carried out in an artificial climate chamber (temperature 23 ± 2 °C, light:dark = 16:8 h, humidity 75% RH). The food was tea leaves (Shuchazao, *Camellia sinensis*) from the Agricultural Development Park of Anhui Agricultural University.

#### 4.4.2. Evaluation of the Stomach Toxicity of *Camellia* Saponins on *E. obliqua*

The leaf-dip bioassay method described by Beloti et al. [42] and Liang et al. [43] was adopted to test the toxicity of tea saponins to second instar *E. obliqua* larvae. Eight tea saponins concentrations (0.5, 15, 25, 40, 50, 80, 100 and 120 mg/mL) were assayed. Distilled water was used to prepare the dilutions. Tea leaf discs (diameter 4 cm) were dipped for 20 s in one of the concentrations. The leaf discs were dried by placing them in a glass Petri dish (diameter 9 cm). Leaf discs in the control group were dipped in distilled water as described above. Thirty *E. obliqua* larvae were starved for 24 h and then transferred to the glass Petri dish (two leaves per Petri dish). Three replicates were made for each concentration. Larvae were considered to be dead if they did not respond when lightly prodded with a brush. Mortality was observed 48 h after treatment and used to establish the respective concentration–response curves.

#### 4.4.3. Midgut Slices of *E. obliqua*

The *E. obliqua* were fed using a leaf soaked with 50 ppm tea saponins according to the method of evaluation of stomach toxicity. After 24 h, the larvae were anesthetized in diethyl ether before the intestines were taken out and fixed in Bouns solution for 12 h. Following this, the intestines were desiccated and diaphanized in alcohol/xylene (1:1) according to Michalany (1980). After placing in paraffin, the samples were sliced in 7-μm sections and stained with hematoxylin–eosin. Morphological alterations of the mid-gut of treated larvae were desiccated and compared to the tissues taken from the control group that were fed untreated leaves and prepared in the same way.

#### 4.4.4. Scanning Electron Microscopy

Water and tea saponins in water (10 mg/L; 1 μL either solution) were separately dropped onto the back of the *E. obliqua* larvae using a Burkard automated microtiter. After 24 h, samples were fixed in 2.5% glutaraldehyde overnight and then washed 4 times with phosphate buffer for 30 min. The samples were dehydrated in a stepwise manner for 30 min in 30%, 50%, 70%, 80%, 90%, and 100% alcohol. Isoamyl acetate was replaced twice for 30 min each time. The samples were dried under vacuum (0.1 mbar, temperature −42 °C). Samples were adhered to the sample plate with conductive tape and observed using a scanning electron microscope (Hitachi SU-8100, Tokyo, Japan) to evaluate the surface structure, shape, and size characteristics of the *E. obliqua*. The SEM was carried out at magnification range of 100–5000×.

#### 4.4.5. Chitin Staining of *E. obliqua* Sections

*E. obliqua* larvae (treated with saponin as described in Section 4.4.4) were fixed overnight in 2.5% glutaraldehyde, washed three times for 5 min each with 150 mM NaCl, 10 mM Na_2_HPO_4_, and 10 mM NaH_2_PO4 (pH 7.2), and then fixed in 30% sucrose glutaraldehyde for 8 h. The anterior of the larvae was embedded in optimum cutting temperature compound (O.C.T. Sakura, Torrance, CA, USA). Sections (8 μm) were cut using a Leica CM1950 microtome (Leica Microsystems, Wetzlar, Germany) and washed three times for 10 min in PBS (137 mM NaCl, 2.7 mM KCl, 10 mM Na_2_HPO_4_, 2 mM KH_2_PO_4_, pH 7.4). The sections were incubated with DAPI dihydrochloride solution (Beyotime), washed three times for 10 min with PBST (PBS containing 0.1% Tween), incubated with wheat germ agglutinin FITC-labeled (WAG) (Sigma), and then washed three times for 10 min with PBST. The slides were photographed using an Olympus BX51 microscope.

### 4.5. Data Analysis

All experiments were carried out at least three times and the data were reported as means and standard deviations. Statistical analyses were performed using SPSS 20.0 (IBM Corp Version 20.0, IBM SPSS Statistics for Windows; IBM, Armonk, NY, USA) and Prism 5 (GraphPad Software, La Jolla, CA, USA) software. Significance was analyzed by the least significant difference (LSD) test with a 95% confidence level (*p* < 0.05).

## Figures and Tables

**Table 1:** Stomach toxicity (*Ectropis obliqua* larvae) of different saponin extracts from *C*. *oleifera* seed cake.

Pesticides	Toxicity Regression Equation ^a^	LC_50_ (mg/L) ^b^	95% Confidence Interval ^c^	R^2^
*C*. *oleifera* seed cake ethanol extract	*y* = −6.69 + 4.12*x*	49.100	39.729–60.126	0.874
30% ethanol eluate	*y* = −6.4 + 3.84*x*	53.239	42.799–66.291	0.862
50% ethanol eluate	*y* = −6.72 + 4.21*x*	45.287	36.527–55.263	0.905
70% ethanol eluate	y = −4.52 + 3.39*x*	22.395	16.489–28.338	0.943

a. Toxicity regression equation represents the relationship between log doses and lethality values. b. LC_50_ represents lethal concentration 50%, the dose required to kill half the members of a tested population after a specified test duration. c. 95% confidence interval of LC_50_.

**Table 2:** Contact toxicity (*Ectropis obliqua* larvae) of different saponin extracts from *C*. *oleifera* seed cake.

Pesticides	Toxicity Regression Equation ^a^	LC_50_ (mg/L) ^b^	95% Confidence Interval ^c^	R^2^
*C*. *oleifera* seed cake ethanol extract	*y* = −5.00 + 3.43*x*	27.380	21.428–36.969	0.977
30% ethanol eluate	*y* = −6.46 + 4.94*x*	21.004	16.848–25.238	0.907
50% ethanol eluate	*y* = −3.75 + 3.14*x*	15.732	8.677–20.814	0.834
70% ethanol eluate	*y* = −3.72 + 4.13*x*	8.459	3.564–11.646	0.984

a. Toxicity regression equation represents the relationship between log doses and lethality values. b. LC_50_ represents lethal concentration 50%, the dose required to kill half the members of a tested population after a specified test duration. c. 95% confidence interval of LC_50_.

## Supplementary Materials

The following are available online, Table S1. The molecular formula of the main compound in 70% ethanol solution and Peak identity (LC/TOF-MS). Table S2. Estimated molecular weights for the main compound in 70% ethanol solution (LC/TOF-MS). Figure S1. Thin layer chromatography analysis of tea saponin components in different eluents. Figure S2. Liquid chromatographic analysis of tea saponins in different eluents. Figure S3. Total ion chromatogram of saponin in 70% ethanol solution (LC/TOF-MS).
