# Supplementary material for: Insecticidal Activity and Insecticidal Mechanism of Total Saponins from Camellia oleifera
Source: Molecules. 2019 Dec 10;24(24):4518. doi: 10.3390/molecules24244518 (PMC6943515; doi:10.3390/molecules24244518)
Supplement: Supplementary file 1 [file molecules-24-04518-s001.pdf]

Supporting Information

Insecticidal activity and insecticidal mechanism of total saponins from *Camellia oleifera*

Chuanjian Cui #[1], Yunqiu Yang#[1] Tianyu Zhao[1], Kangkang Zou[1], Chuanyi Peng[1], Huimei Cai[1], Xiaochun Wan[1] and Ruyan Hou [1,*]

[1] State Key Laboratory of Tea Plant Biology and Utilization School of Tea and Food Science & Technology, Anhui Agricultural University, Hefei, 230036, China; E-Mails: 63667074@qq.com (C.C.J); yangyunqiu@ahau.edu.cn (Y.Q.Y); 276708894@qq.com (T.Y.Z); 792493250@qq.com (K.K.Z); pcy0917@ahau.edu.cn (C.Y.P) ; hml20@sina.com (H.M.C); xcwan@ahau.edu.cn (X.C.W) ;

[2] Affiliation 2; e-mail@e-mail.com

***** Correspondence: hry@ahau.edu.cn; Tel.: Tel: +86 -551-65786765

[#] Chuanjian Cui and Yunqiu Yang contributed equally to this work.

Supporting information:

Table S1. The molecular formula of the main compound in 70% ethanol eluate and Peak identity (LC / TOF-MS).

Table S2. Estimated molecular weights for the main compound in 70% ethanol eluate (LC/TOF-MS).

Figure S1. Thin layer chromatography analysis of Tea saponin components in different eluents.

Figure S2. Liquid chromatographic analysis of Tea saponin in different eluents.

Figure S3. Total ion chromatogram of saponin in 70% ethanol eluate (LC/TOF-MS).

Table S1. The molecular formula of the main compound in 70% ethanol eluate and Peak identity (LC / TOF-MS).

**Peak**	**Retention Time (min)**	**[M−H] −**	**MS^2**	**Peak**	**Retention Time (min)**	**[M−H] −**	**MS^2**
**1**	4.834	1201.5933	1021.5729	**16**	13.733	1187.5875	1035.5285
	6.209	1233.5984	1053.5253	**17**	14.959	1215.5823	1035.5144
**3**	6.590	1203.5851	969.4664	**18**	16.285	1245.5941	1065.5355
**4**	7.485	1189.5717	1009.5066	**19**	17.014	1215.5832	1035.5144
**5**	7.800	1293.6147	1071.5402	**20**	18.406	1287.6083	1085.5317
**6**	8.181	1263.6038	1081.5292	**21**	18.787	1289.6195	1085.5713
**7**	8.844	1261.5871	1081.5286	**22**	20.179	1301.6210	1119.5499
**8**	9.407	1231.5780	1051.5206	**23**	20.991	1301.6197	1121.5555
**9**	9.938	1201.5658	1021.889	**24**	21.190	1299.6041	1119.5363
**10**	10.219	1233.5932	1053.5253	**25**	21.555	1303.6357	921.4911
**11**	10.766	1203.5826	969.4664	**26**	22.085	1299.6041	1119.5363
**12**	11.148	1201.5674	1132.5289	**27**	22.599	1301.6196	1119.5499
**13**	12.556	1223.5588	1044.5006	**28**	26.360	1347.6048	1135.5608
**14**	12.871	1171.5604	1043.4828	**29**	26.775	1313.6200	1181.5612
**15**	13.219	1217.5976	1143.5642				

Table S2. Estimated molecular weights for the main compound in 70% ethanol eluate (LC/TOF-MS).

Peak	Formula	Peak identity	Peak	Formula	Peak identity
**1**	$C_{58}H_{90}O_{26}$	Camelliasaponin B2	**16**	$C_{58}H_{92}O_{25}$	Camelliasaponin A1
**2**	$C_{58}H_{90}O_{28}$	Unknown	**17**	$C_{59}H_{92}O_{26}$	Oleiferasaponin A1
**3**	$C_{58}H_{92}O_{26}$	Theasaponin H1	**18**	$C_{60}H_{94}O_{2}$	Oleiferasaponin C4
**4**	$C_{56}H_{82}O_{21}$	Unknown	**19**	$C_{59}H_{92}O_{26}$	Oleiferasaponin C1
**5**	$C_{62}H_{98}O_{25}$	Unknown	**20**	$C_{62}H_{96}O_{28}$	Teaseedsaponin A
**6**	$C_{63}H_{92}O_{26}$	Oleiferasaponin C3	**21**	$C_{62}H_{98}O_{28}$	Unknown
**7**	$C_{63}H_{90}O_{26}$	Unknown	**22**	$C_{63}H_{98}O_{28}$	Teaseedsaponin D
**8**	$C_{59}H_{92}O_{27}$	Theasaponin A2	**23**	$C_{62}H_{94}O_{29}$	Theasaponin E11
**9**	$C_{58}H_{90}O_{26}$	Camelliasaponin B1	**24**	$C_{63}H_{96}O_{28}$	Teaseedsaponin I
**10**	$C_{58}H_{90}O_{28}$	Unknown	**25**	$C_{62}H_{96}O_{29}$	Theasaponin A7
**11**	$C_{58}H_{92}O_{26}$	Camelliasaponin C1	**26**	$C_{63}H_{96}O_{28}$	Oleiferoside L
**12**	$C_{58}H_{92}O_{26}$	Camelliasaponin C2	**27**	$C_{63}H_{98}O_{28}$	Oleiferoside J
**13**	$C_{58}H_{96}O_{27}$	Unknown	**28**	$C_{64}H_{100}O_{30}$	Unknown
**14**	$C_{57}H_{88}O_{25}$	Assamsaponin A	**29**	$C_{63}H_{94}O_{29}$	Unknown
**15**	$C_{58}H_{90}O_{27}$	Theasaponin F1			

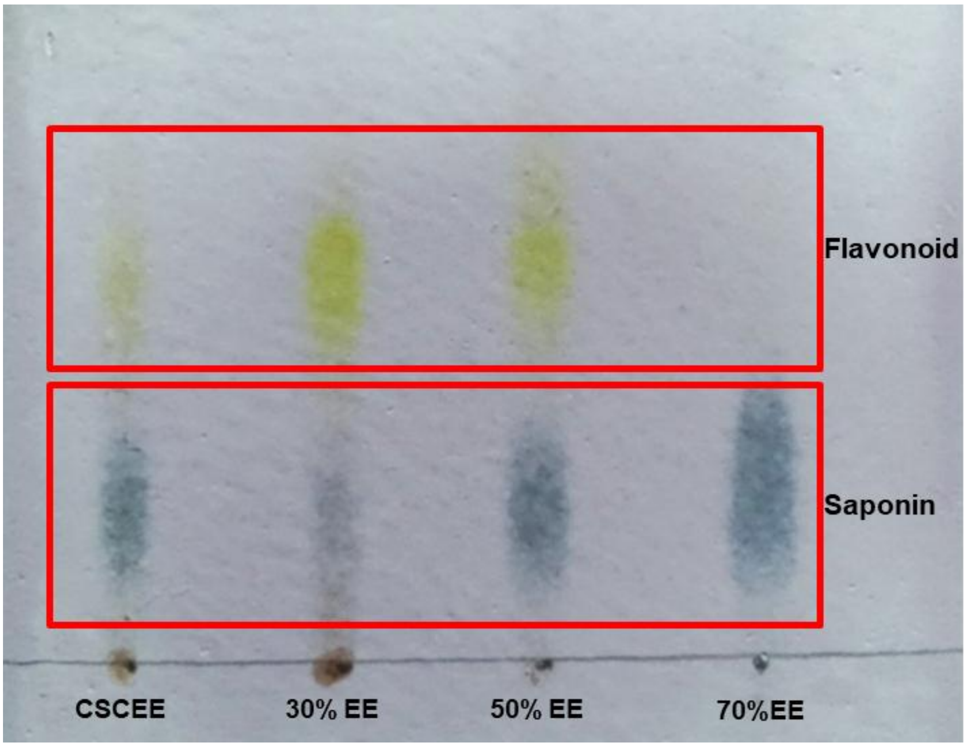

Figure S1. Thin layer chromatography analysis of tea saponin components in different eluents (CSCEE: *C. oleifera* seed cake ethanol extract; 30% EE: 30% ethanol eluate; 50% EE: 50% ethanol eluate; 70% EE: 70% ethanol eluate).

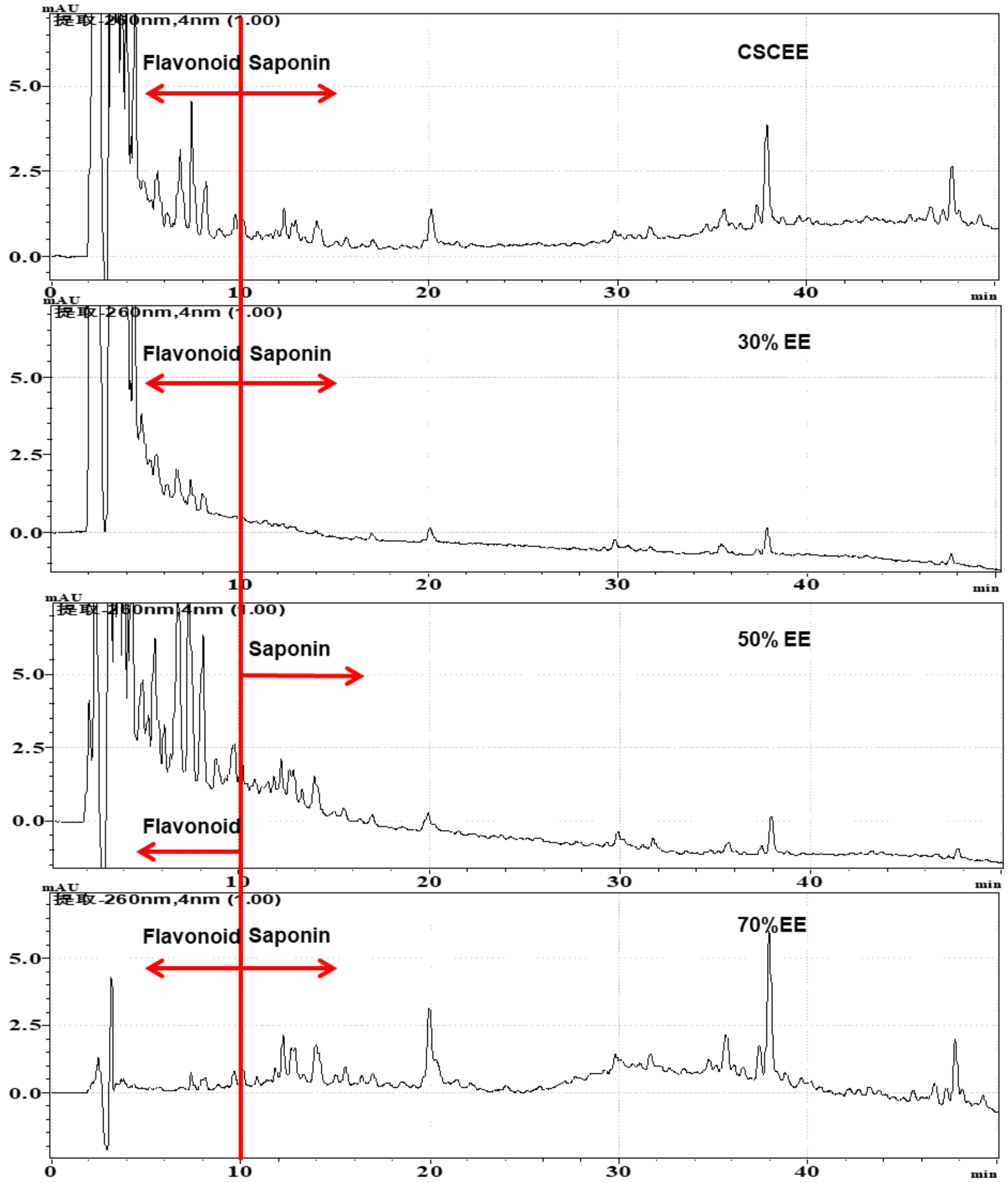

Figure S2. Liquid chromatographic analysis of tea saponins in different eluents (CSCEE: *C. oleifera* seed cake ethanol extract; 30% EE: 30% ethanol eluate; 50% EE: 50% ethanol eluate; 70% EE: 70% ethanol eluate).

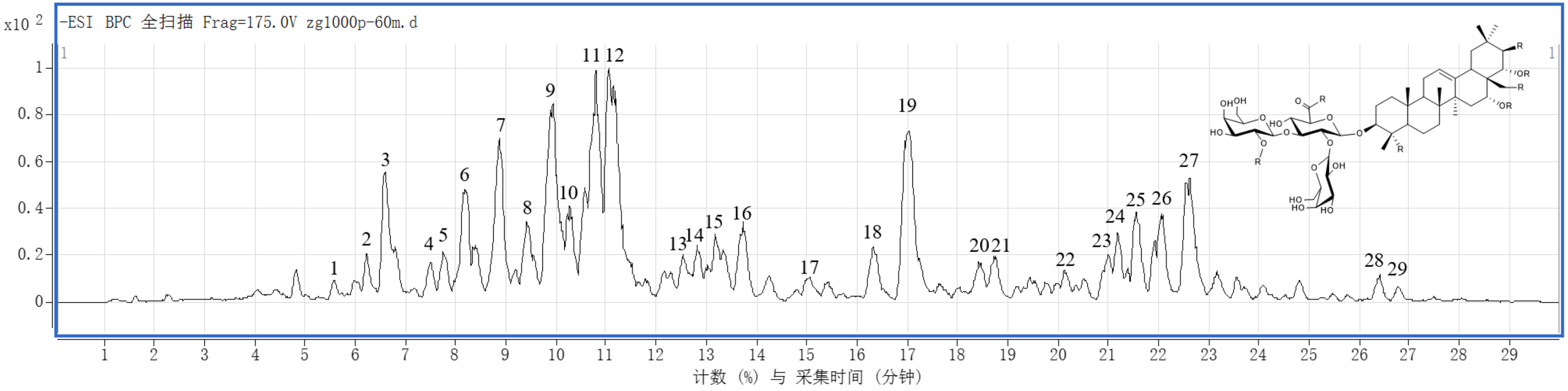

Figure S3. Total ion chromatogram of saponin in 70% ethanol eluate (LC/TOF-MS)
